# Supplementary material for: Experimental and Numerical Study of Al2219 Powders Deposition on Al2219-T6 Substrate by Cold Spray: Effects of Spray Angle, Traverse Speed, and Standoff Distance
Source: Materials (Basel). 2023 Jul 26;16(15):5240. doi: 10.3390/ma16155240 (PMC10419605; doi:10.3390/ma16155240)
Supplement: Supplementary file 1 [file materials-16-05240-s001.zip › materials-2504101-supplementary.pdf]

Supplementary Information for "Experimental and Numerical Study of Al2219 Powders Deposition on Al2219-T6 Substrate by Cold Spray: Effects of Spray Angle, Traverse Speed and Standoff Distance"

Zheng ZHANG,[a] Tzee Luai MENG,[a] Coryl Jing Jun LEE,[a] Fengxia WEI,[a] Te BA,[b#] Zhi-qian ZHANG[b‡] and Jisheng PAN [a+]*

Corresponding Authors:

[*]Tel.: +65-64168974
[*]E-mail address: zhangz@imre.a-star.edu.sg

[#]Tel.: +65-64191560
[#]E-mail address: bat@ihpc.a-star.edu.sg

[‡]Tel.: +65-64191535
[‡]E-mail address: zhangz@ihpc.a-star.edu.sg

[+]Tel.: +65-63194919
[+]E-mail address: js-pan@imre.a-star.edu.sg

1. Images of the nine sets of Al2219 deposits at three transverse speed and three stand-off distance with seven spray angles

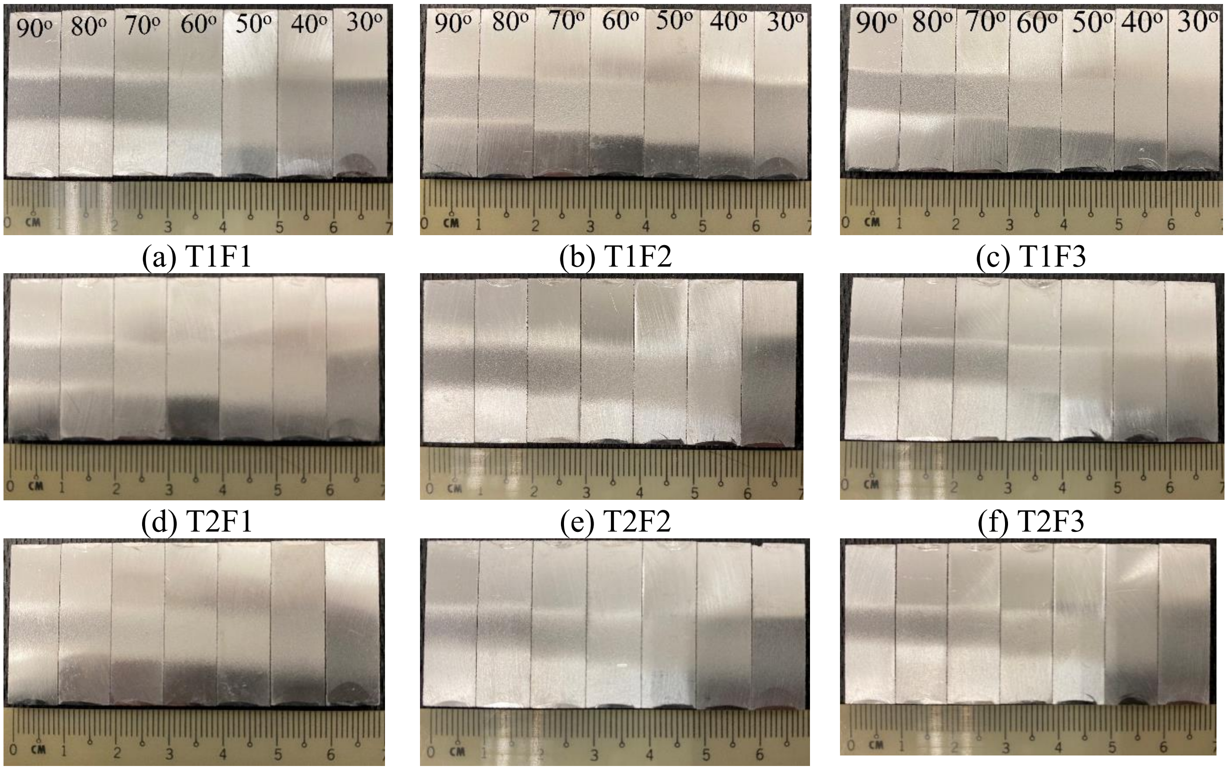

(a) T1F1 (b) T1F2 (c) T1F3

(d) T2F1 (e) T2F2 (f) T2F3

(g) T3F1 (h) T3F2 (i) T3F3

Figure S1. *Pictures of nine sets of Al2219 coupons deposited at three transverse speed and three stand-off distance with seven spray angles from 90 (far left) to 30°(far right).*

2. Surface morphology of Al2219 deposits by SEM at three traverse speed and three stand-off distance with seven spray angles

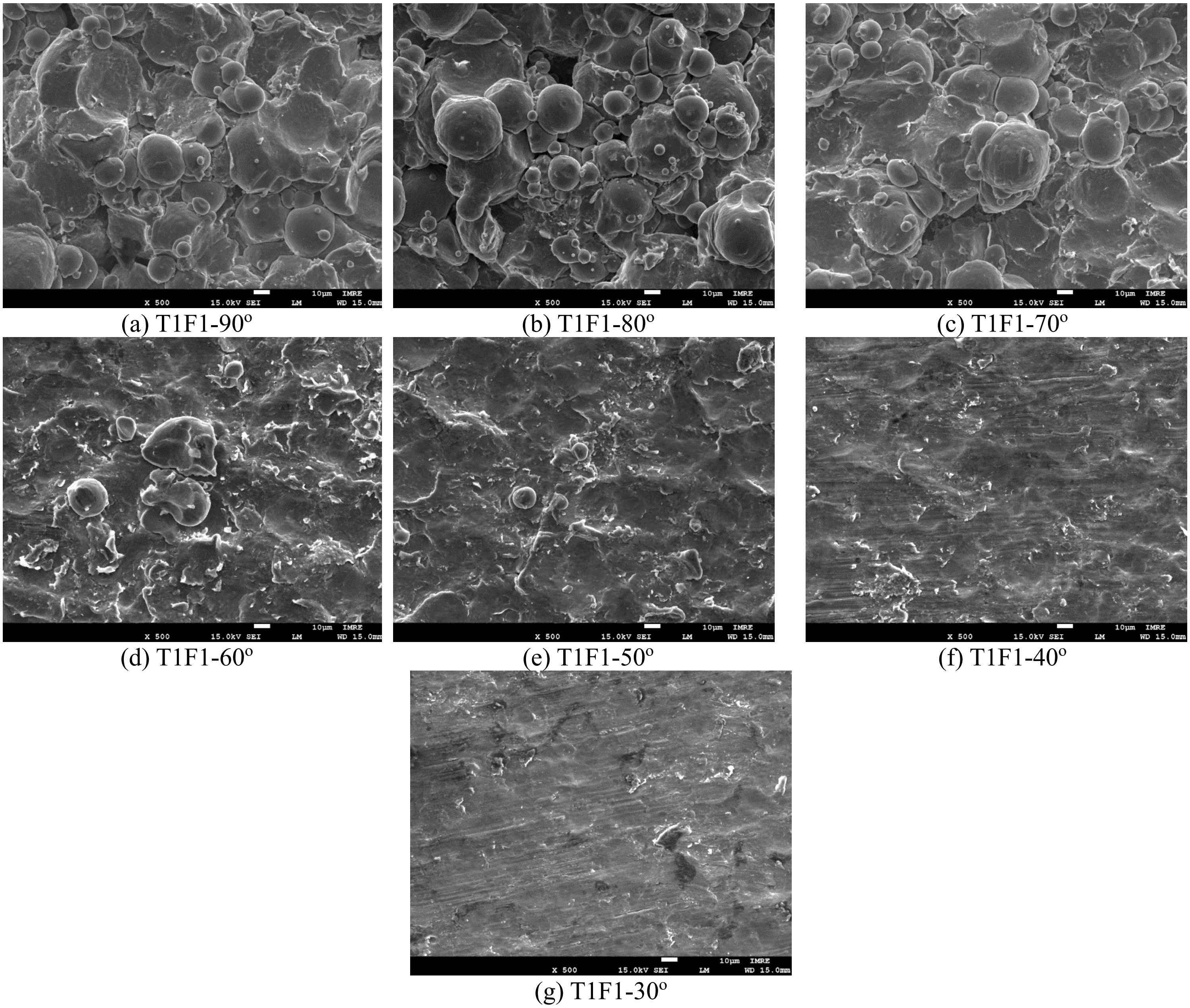

Figure S2. *Surface morphology of Al2219 deposits by SEM after spraying at seven spray angles at T1F1 condition (i.e., at the traverse speed of 200 mm/s (T1) and the standoff distance of 9 cm (F1)).*

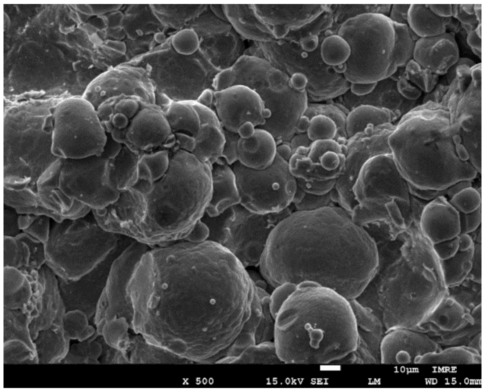

(a) T1F2-90º

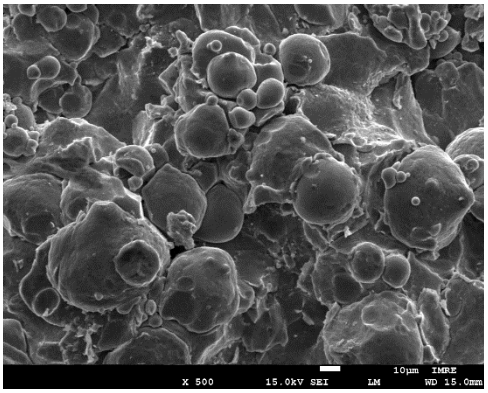

(b) T1F2-80º

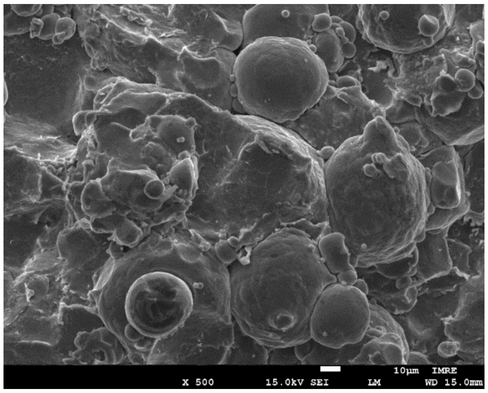

(c) T1F2-70º

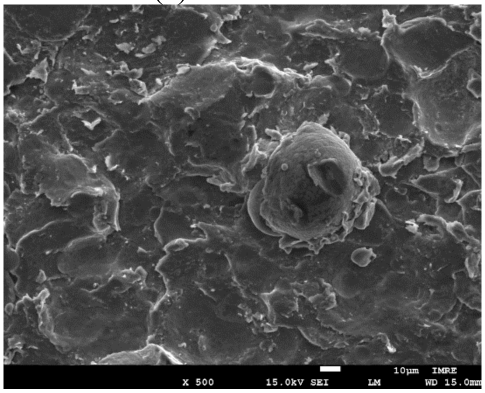

(d) T1F2-60º

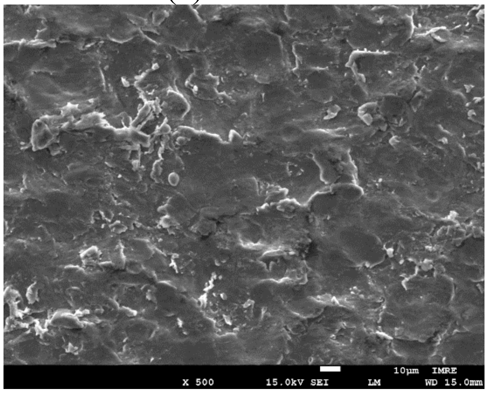

(e) T1F2-50º

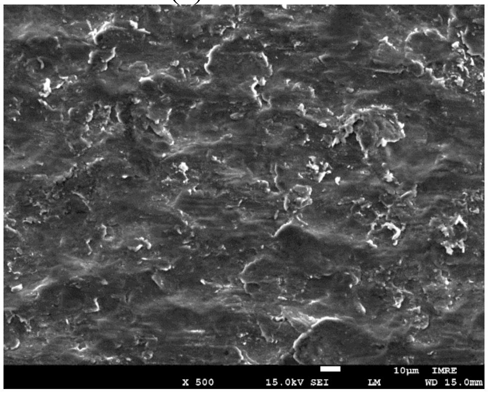

(f) T1F2-40º

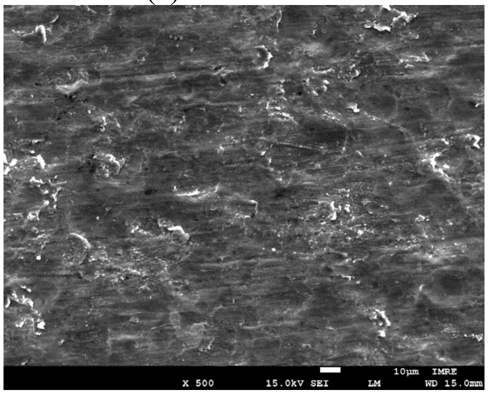

(g) T1F2-30º

Figure S3. *Surface morphology of Al2219 deposits by SEM after spraying at seven spray angles at T1F2 condition (i.e., at the traverse speed of 200 mm/s (T1) and the standoff distance of 6 cm (F2)).*

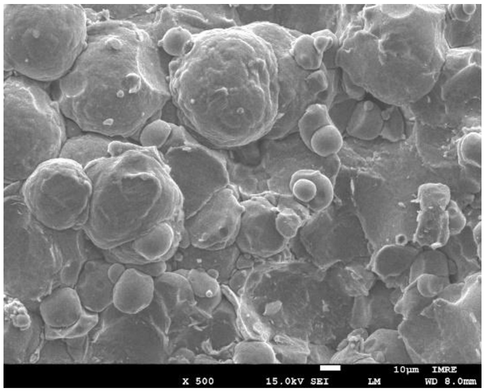

(a) T2F1-90º

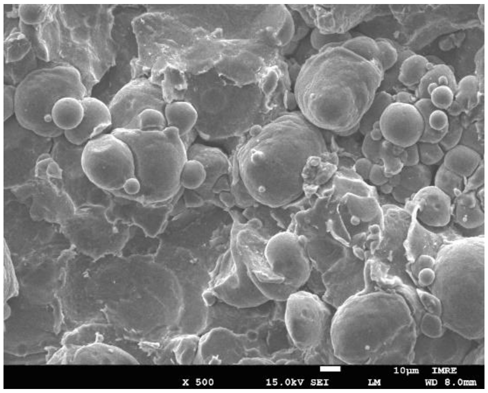

(b) T2F1-80º

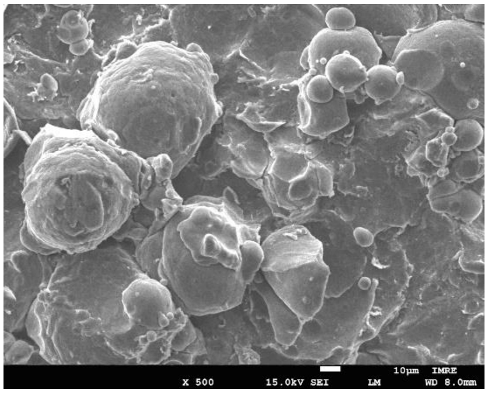

(c) T2F1-70º

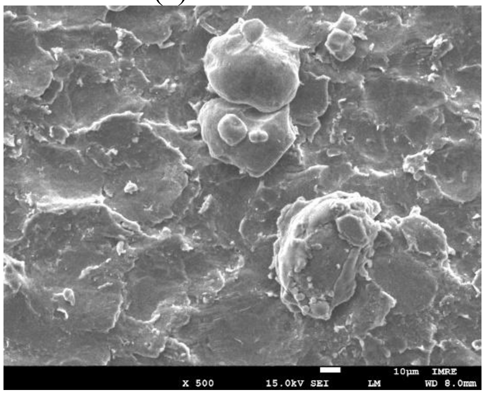

(d) T2F1-60º

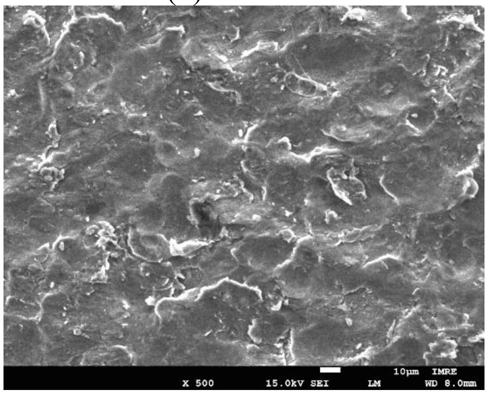

(e) T2F1-50º

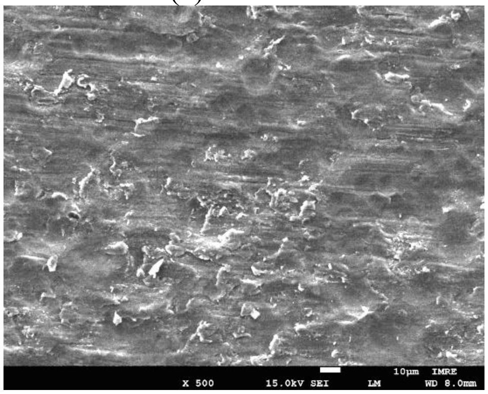

(f) T2F1-40º

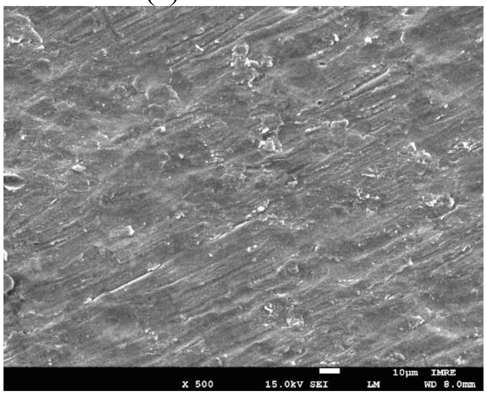

(g) T2F1-30º

Figure S4. *Surface morphology of Al2219 deposits by SEM after spraying at seven spray angles at T2F1 condition (i.e., at the traverse speed of 350 mm/s (T2) and the standoff distance of 9 cm (F1)).*

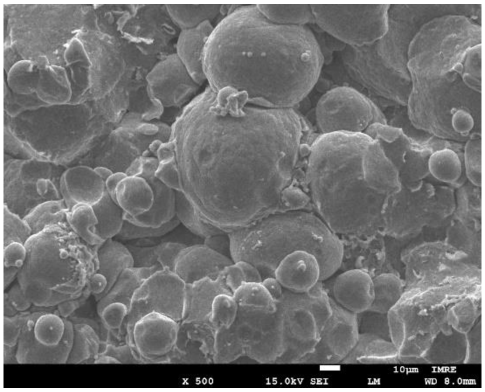

(a) T2F2-90º

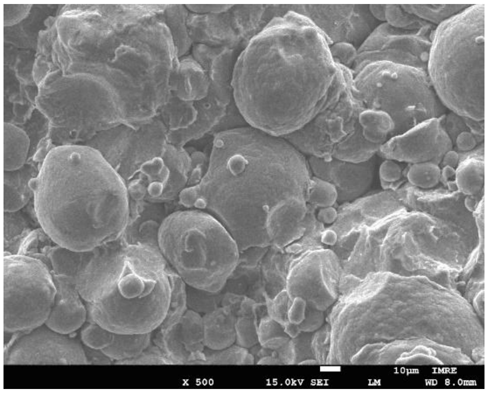

(b) T2F2-80º

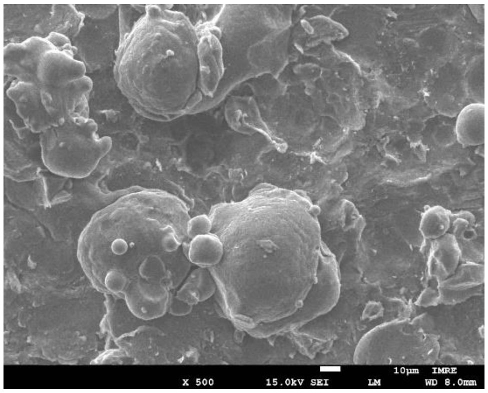

(c) T2F2-70º

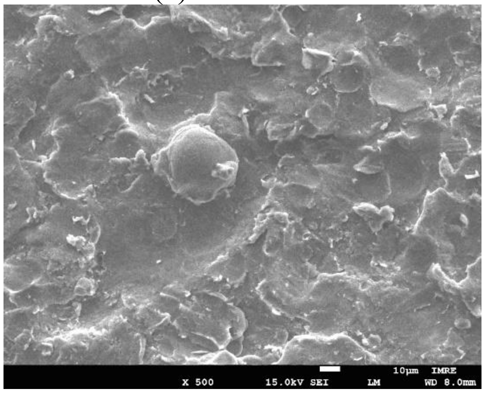

(d) T2F2-60º

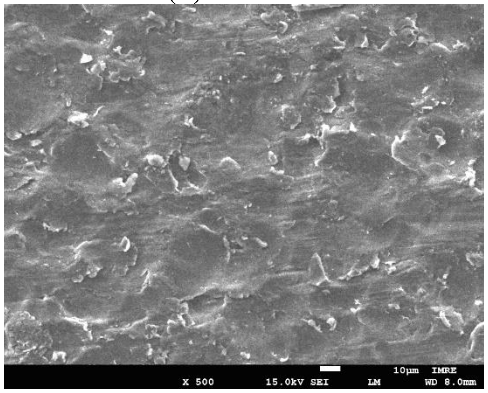

(e) T2F2-50º

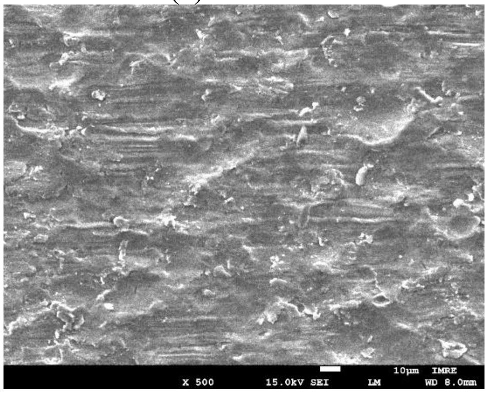

(f) T2F2-40º

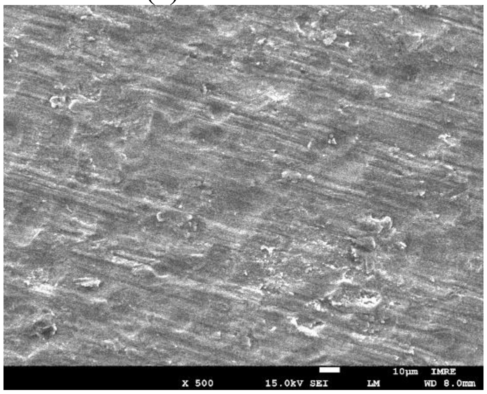

(g) T2F2-30º

Figure S5. *Surface morphology of Al2219 deposits by SEM after spraying at seven spray angles at T2F2 condition (i.e., at the traverse speed of 350 mm/s (T2) and the standoff distance of 6 cm (F2)).*

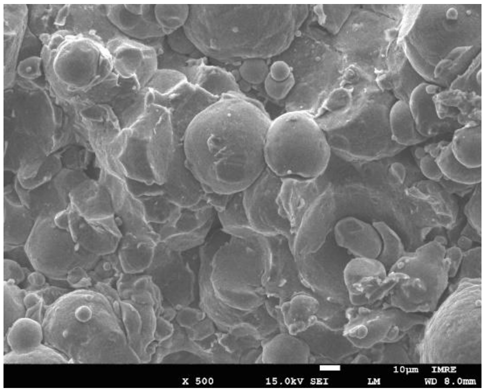

(a) T2F3-90º

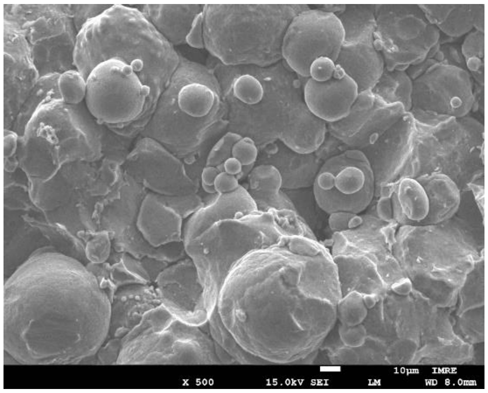

(b) T2F3-80º

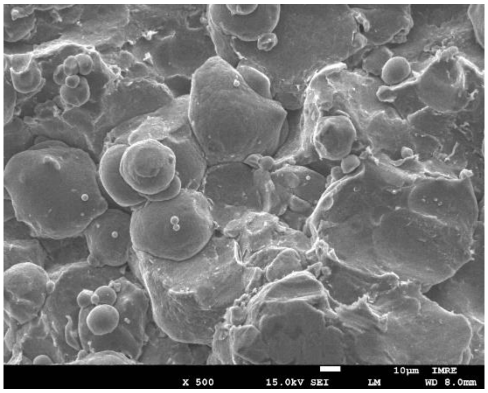

(c) T2F3-70º

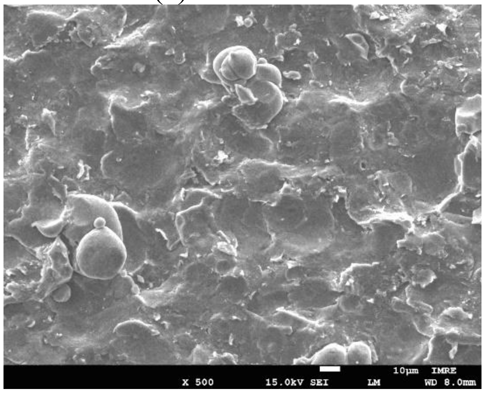

(d) T2F3-60º

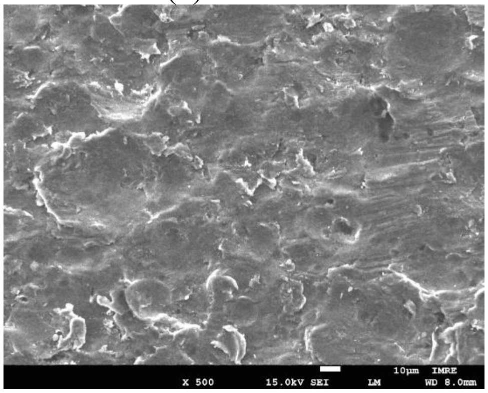

(e) T2F3-50º

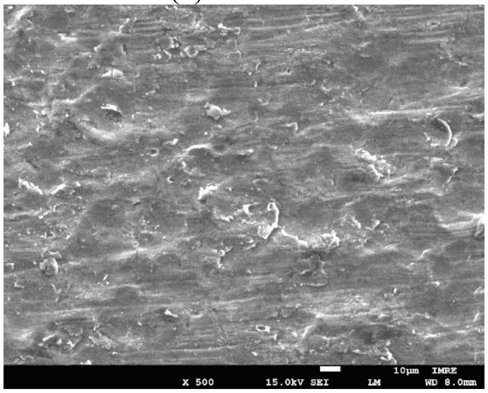

(f) T2F3-40º

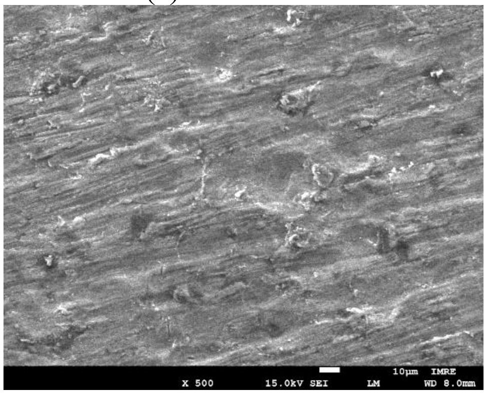

(g) T2F3-30º

Figure S6. *Surface morphology of Al2219 deposits by SEM after spraying at seven spray angles at T2F3 condition (i.e., at the traverse speed of 350 mm/s (T2) and the standoff distance of 3 cm (F3)).*

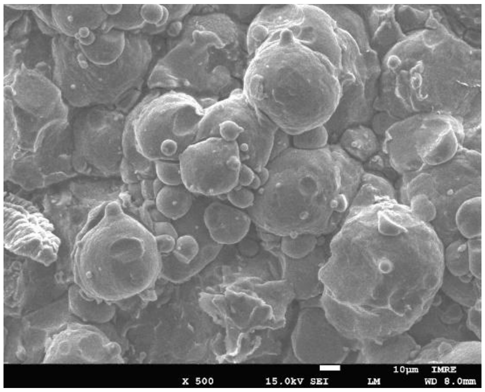

(a) T3F2-90º

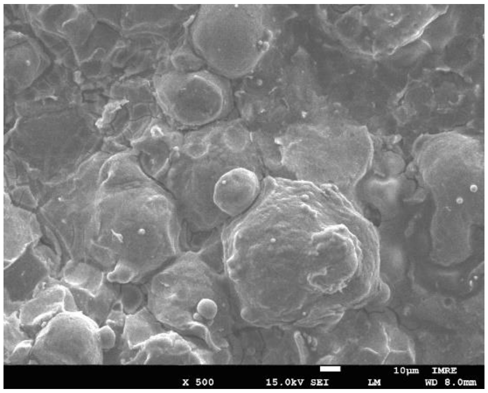

(b) T3F2-80º

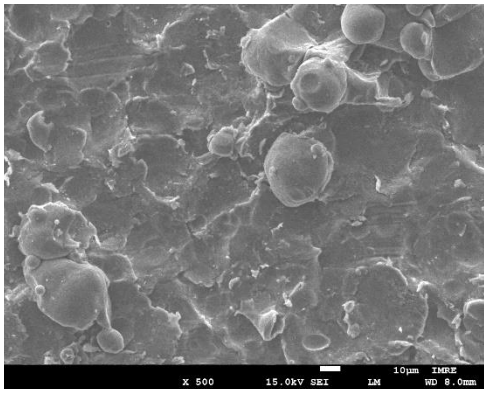

(c) T3F2-70º

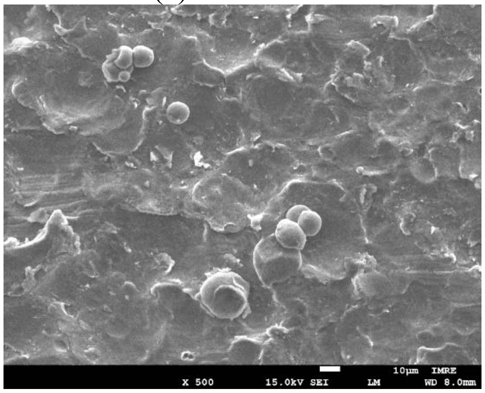

(d) T3F2-60º

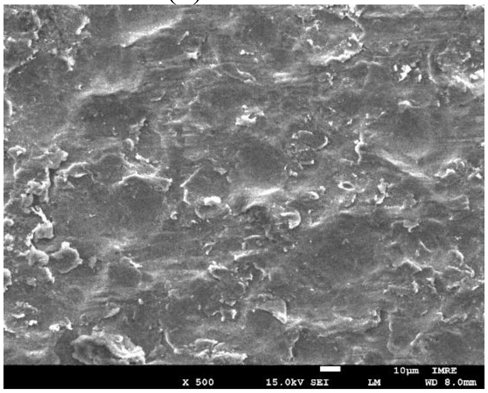

(e) T3F2-50º

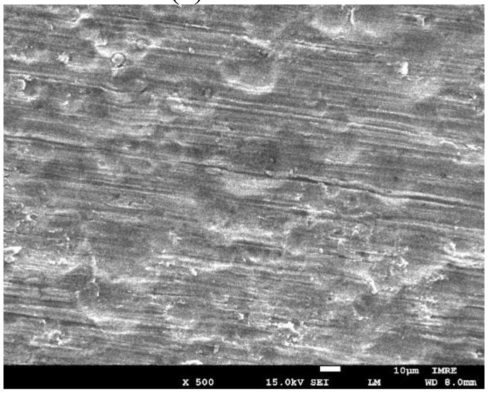

(f) T3F2-40º

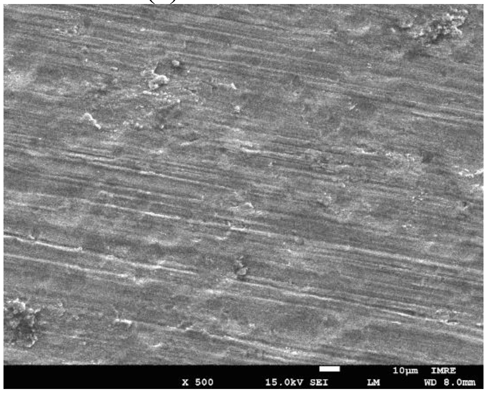

(g) T3F2-30º

Figure S7. *Surface morphology of Al2219 deposits by SEM after spraying at seven spray angles at T3F2 condition (i.e., at the traverse speed of 500 mm/s (T3) and the standoff distance of 6 cm (F2)).*

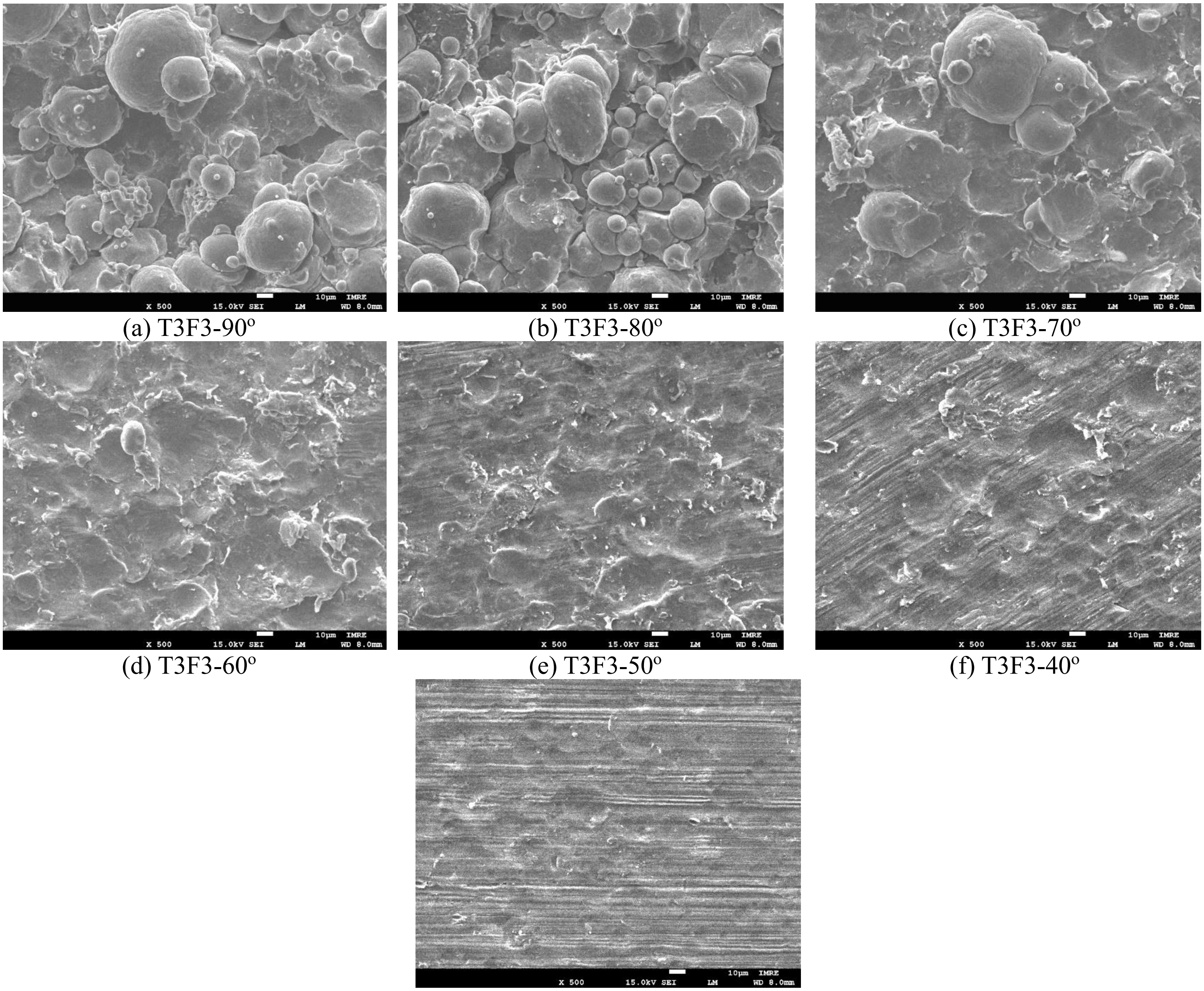

(a) T3F3-90º (b) T3F3-80º (c) T3F3-70º

(d) T3F3-60º (e) T3F3-50º (f) T3F3-40º

(g) T3F3-30º

Figure S8. *Surface morphology of Al2219 deposits by SEM after spraying at seven spray angles at T3F3 condition (i.e., at the traverse speed of 500 mm/s (T3) and the standoff distance of 3 cm (F3)).*

3. Details of Finite Element Model (FEM) for identifying critical angle for bonding

The FEM model is shown in Fig. S9, which is a 1/2 symmetric model, which is developed based on the commercial FEM software package Abaqus/Explicit in the Eulerian framework. Adiabatic

condition is assumed in this single particle impact simulation. The regular Eulerian mesh with mesh size D/40 in the vicinity of the particle is applied, where D is the diameter of the particle. The substrate size is 4D in x direction, 2D in y and z directions as shown in Fig. S9. The element type is EC3D8R, which is three-dimensional, 8-node thermally coupled element using reduced integration for explicit Eulerian analysis. The particle is assumed to be in ideal sphere shape with an average diameter 43.8 μm, consistent with the D_{50} particle diameter used in the experiment. The initial temperature of the powder particles is 460K which was simulated by the CFD modeling. The initial temperature of the substrate is assumed to be 300K. This setting is to simulate the first layer bonding. In the FEM mode, the symmetric boundary conditions and iso-thermal condition are applied on the symmetric plane, and non-reflecting boundary condition and constant temperature 300K condition are imposed on other surface planes of the domain, in order to mimic the semi-infinite body of the substrate and eliminate the boundary effect in the simulations.

The constitutive material models for both Al2219 powder particle and substrate employ Johnson-Cook plasticity model given by Eq.(1). The material properties used for the simulation modeling are shown in Table 2. The elastic behavior of the materials was modelled by inputting Young's modulus and Poisson ratio based on the material properties of Al2219.

The Al2219 powder particle's initial temperature and initial impact velocity magnitude are 300 K and 620 m/s, respectively. 620 m/s is also the critical velocity for bonding and the average particle velocity measured in the experiment. The Al2219 substrate's initial temperature and initial velocity are 300 K and zero (stationary), respectively. The non-reflect boundary condition is imposed on the side and bottom surfaces of the substrate to model the semi-infinite substrate condition.

The simulations study different impact angle θ. In the simulation, the impact starts from time $t = 0$, and stops at 1ms. If the particle adheres to the substrate, the kinetic energy would be dissipated and the particle velocity would decrease to a very small value close to zero, as shown in Fig. 9 ($\geq 60^o$). If the particle does not adhere, the particle would slide along the substrate surface with high velocity and finally move out of the simulation domain. As a result, these particles are not shown in the simulation frame, such as the results with small impact angle (50 and 45°) shown in Fig. 9.

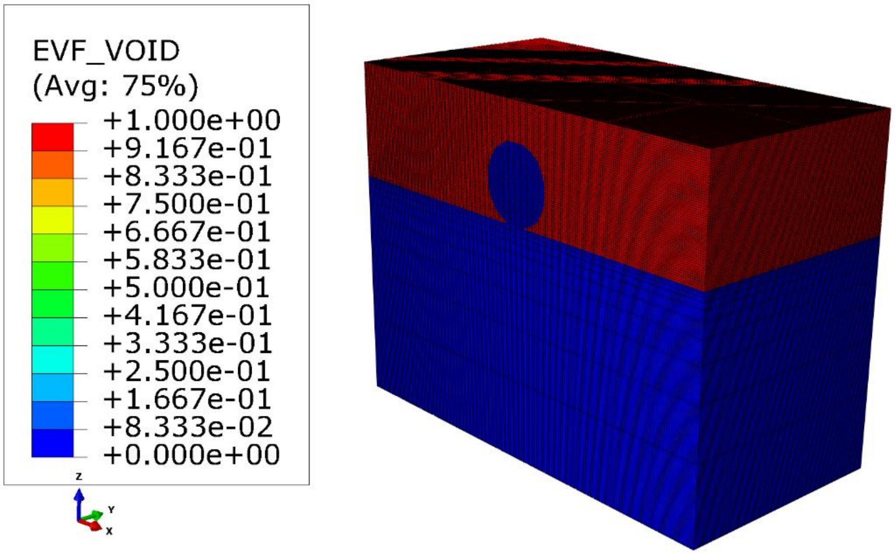

Figure S9. *A 3D single-particle 1/2 –symmetric model based on FEM in Eulerian framework using Abaqus/Explicit. The blue colour indicates the elements filled with the material, while the red color indicates the void (i.e., there is no material in the element).*

Using the Finite Element Method (FEM) in the Eulerian framework provided by Abaqus/Explicit, the two materials (i.e., particle material and substrate material) will be assumed to be adhered if the two materials fill in one element. However, in the scenario of the high-velocity impact between particle and substrate in the cold spray process, extremely large plastic deformation will occur, converting the kinetic energy of the particle materials into the plastic strain energy and thermal energy. The heat generated within a very short time (less than 100ns) after the impact will elevate the temperature of the material, especially in the thin layer (the thickness is determined by the element size) of the materials at the particle-substrate interface. The temperature of the interface will reach the melting point or close to the melting point. The thermal softening of the material will be significant at the particle-substrate interface, and the material will lose its strength and behave like a viscous "fluid". This phenomenon is governed by the Johnson-Cook plasticity material model.

When the particle impacts at a small impact angle (≤55°), the particle cannot be adhered due to the loss of the strength of the interface material. The thermal-softened material's strength is small and the velocity component parallel to the target surface is large. Therefore, the particle will slide away or rebound after impact in a very short time. In this regime, the particle will not adhere on the substrate.

When the particle impacts at a large impact angle (≥ 60°), the particle can adhere even if the interface material is thermally softened. This is due to the large velocity component of the particle perpendicular to the target surface creating a deep crater. Hence, the particle cannot slide away because of the small velocity component parallel to the target surface. Therefore, the particle will stay inside the crater. After cooling down, the material interface material recovers its strength.

4. Details of Finite Element Model (FEM) for evaluating coating residual stresses

To study the residual stresses in metal cold spray coatings, the single-particle impact model is not sufficient to address the real-life complex conditions in the metal cold spray process. A multiple particles impact model based on FEM is employed, which was reported in our recent publication [S. Msolli, Z.-Q. Zhang, D.H.L. Seng, Z. Zhang, J. Guo, C.D. Reddy, N. Sridhar, J. Pan, B.H. Tan, Q. Loi, *An experimentally validated dislocation density based computational framework for predicting microstructural evolution in cold spray process*, Int. J. Solids Struct., 225 (2021) 111065]. The material properties of the particles and substrate are the same as the above single-particle impact model given in Section 3. The main difference is the number of particles as shown in Fig. S10.

Several hundreds of particles are distributed above the substrate as illustrated in Fig. S10. The particles are denoted as red color and substrate are denoted in blue color. In the figure, void material is not displayed in order to show clear particle distribution. The particles are distributed in the way that the substrate surface can be fully covered after deposition. The particles are given many layers, and the distance between each layer is the same to the particle diameter. In this simulation, the particles are given the same diameter equal to the average diameter measured by the experiment.

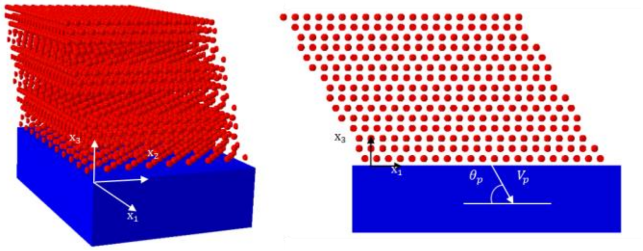

Figure S10. *A 3D FEM model based on Abaqus/Explicit in Eulerian framework. The blue colour indicates the elements filled with the substrate material, and the red colour indicates the elements filled with the substrate.*

The simulations assume thermal-mechanical coupling conditions. Heat conduction is considered. The initial particle temperature is 460K, which is the average particle temperature simulated by CFD model, and the particle velocity magnitude is 620m/s. The substrate's initial temperature is 300K. The side and bottom surfaces of the substrate are imposed by the non-reflect boundary condition provided by Abaqus to mimic the semi-infinite substrate domain.
